# A Simple Colorimetric Method for the Determination of Raloxifene Hydrochloride in Pharmaceuticals Using Modified Romini's Reagent

**DOI:** 10.1155/2019/3021980

**Published:** 2019-12-18

**Authors:** Kovvuru Praneeth Kumar Reddy, Kale Muni Sai Prathap, Hemraj Sharma, Kondareddy Vinod Kumar

**Affiliations:** ^1^Department of Pharmaceutical Analysis and Quality Assurance, Raghavendra Institute of Pharmaceutical Education and Research (RIPER), Anantapur, Andhra Pradesh, India; ^2^Department of Pharmacy, Shree Medical and Technical College, Bharatpur, Nepal

## Abstract

An analytical method has been developed based on a colorimetric assay for the estimation of raloxifene hydrochloride, followed by validation of the optimized method by using the ICH guidelines. The new method, aromatic ring derivatization technique, is based on a coupling reaction using modified Romini's reagent in which sodium nitroprusside is used as a chromogenic derivatizing reagent. Raloxifene contains a phenolic hydroxyl group, containing reactive hydrogen. This reactive proton reacts with sodium nitroprusside and reduces it to sodium meta-hydrogen ferrocyano nitrate, which is a colored product. Optimization studies revealed that the coupling reaction was very rapid and completed in less than 1 minute. A 1 : 1 drug-to-reagent stoichiometric ratio was obtained for the azo dye formed. The azo adduct formed exhibits a bathochromic shift with absorption maximum *λ*_max_ at 440 nm, which was selected as the analytical wavelength. The drug seems to be linear, which was established via the regression analysis from 20 to 120*μ*g/ml. LOD and LOQ of the developed method were found to be 1.807*μ*g/ml and 5.47*μ*g/ml, respectively. Interday and intraday precision was studied, and %RSD was less than 2. Since the stability of the drug and the reagent was found to be predominantly massive, this method can be used for the formulation of raloxifene hydrochloride . The method can be extended for the routine assay of raloxifene formulations.

## 1. Introduction

**Figure 1:**
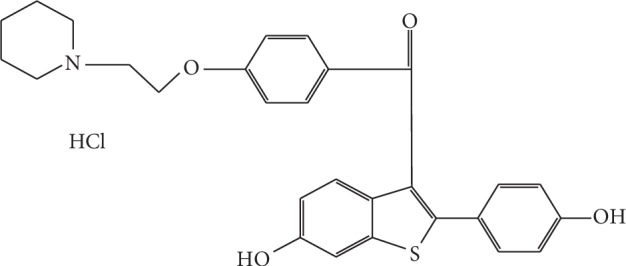
Structure of raloxifene hydrochloride.

Raloxifene hydrochloride [1] is a new antiosteoporotic agent, with the chemical name 6-hydroxy-2-(4-hydroxy phenyl) benzo {b} thien-3-yl] [4-{2-(1-piperidinyl)-ethoxy}-phenyl] methanone (Figure 1). Clinically, it is effective in the treatment of breast cancer [2, 3].

A few UV methods with UV detection have been previously reported for the determination of raloxifene hydrochloride in pharmaceuticals [4–7]. Other techniques reported for the assay of raloxifene in pharmaceuticals include HPLC [8–13], Rayleigh scattering method [14], LC-MS/MS [15], and capillary electrophoresis [16].

This paper deals with the development and validation of a sensitive colorimetric method for the assay of raloxifene hydrochloride in pharmaceuticals. Nowadays, the use of more sophisticated instruments such as HPLC replaces various classical methods of analysis. However, the high attainment and maintenance cost of this equipment makes the implementation rather inconvenient, especially in resource-poor economies. Various UV methods have been developed till date, but many of the methods require the application of heat to stabilize the color. A few methods were found to be less precise and in a few methods the reagents interfered with the drug. Hence, it is necessary to develop an easy and precise method with the long stability of color.

The main purpose of the present study is to establish a relatively simple, sensitive, valid, and inexpensive colorimetric method for the determination of raloxifene hydrochloride in the pure form using modified Romini's reagent which includes sodium nitroprusside and 0.1% zinc chloride with acetone. Since most of the previous methods have been found to be relatively complicated and expensive, there is an obvious demand in developing a method based on the reaction between the drug and sodium nitroprusside and 0.1% zinc chloride in acetone without the effort of high temperature and heat to stabilize the color. The present investigation aims to develop a sensitive and cost-effective method for the determination of raloxifene hydrochloride in the pure form and in the dosage form using the spectrophotometric technique. The proposed method has the advantages of great sensitivity and simplicity along with good accuracy and precision. The method is applied successfully for the determination of raloxifene in the tablet form without the interference of excipients. The color developed was stable for a long period of time; hence, this method can be extended for the routine assay of raloxifene hydrochloride formulations. The method was validated as per the International Conference on Harmonization (ICH) guidelines [17, 18].

## 2. Materials and Methods

A Systronics Digital Colorimeter model-112 with a photodiode detector was used for the measurement of optical density. All the chemicals used were of analytical grade and were freshly prepared.

### 2.1. Preparation of Standard Solutions

A standard stock solution of raloxifene was prepared in methanol (1000 *μ*g/ml) (1^0^ stock). For the primary stock 1–6 ml, the solutions were pipetted, respectively, in six different 50 ml volumetric flasks. To each flask, 5.5 ml of sodium nitroprusside, 0.8 ml of 0.1% zinc chloride, and 1 ml of acetone were added, and the volume was made up to the mark using methanol to get the working samples of 20–120 *μ*g/ml. The absorbance of the colored chromogen was measured at 440 nm against the reagent blank.

### 2.2. Method Development

#### 2.2.1. Determination of Solubility

Raloxifene solubility was tested in different organic and aqueous solvents, and the solubility was found in methanol.

#### 2.2.2. Selection of Suitable Reagent

Modified Romini's reagent was selected. It was composed of modified sodium nitroprusside, saturated aqueous zinc chloride solution, and acetone. The volume of the reagent to be added was optimized.

#### 2.2.3. Preparation of Reagent

Sodium nitroprusside solution: 10 gm of sodium nitroprusside was dissolved in 100 ml of water. Zinc chloride solution: 0.1 gm zinc chloride solution was dissolved in 100 ml of water.

### 2.3. Mechanism of Color Production

**Figure 2:**
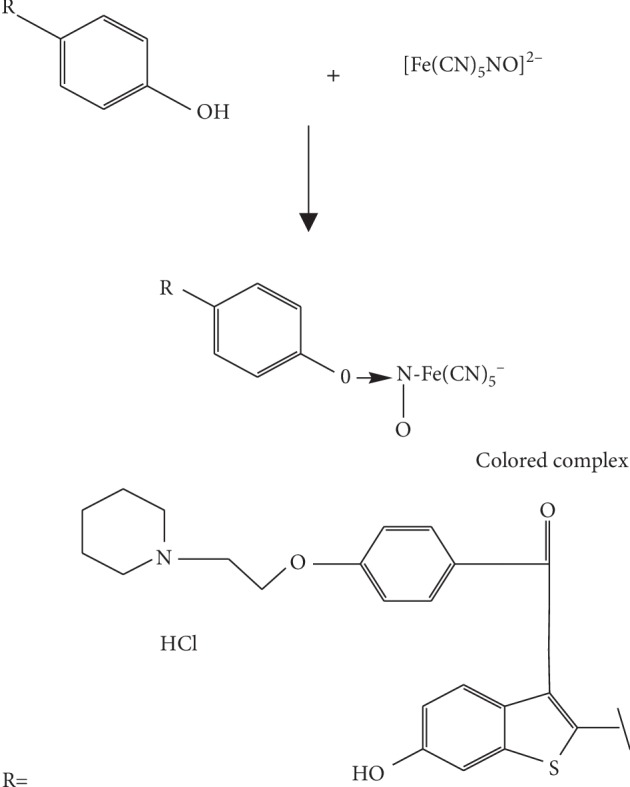
Formation of the colored complex.

Raloxifene contains phenolic hydroxyl group containing reactive hydrogen. This reactive proton reacts with sodium nitroprusside and reduces it to sodium meta-hydrogen ferrocyano nitrate, which is a colored product as shown in Figure 2.

## 3. Results and Discussion

**Figure 3:**
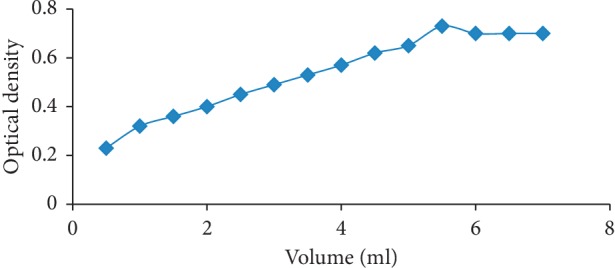
Volume optimization of the modified sodium nitroprusside reagent at 5.5 ml.

Sodium nitroprusside was allowed to react with raloxifene hydrochloride along with zinc chloride and acetone to form a reddish product with absorption maxima at 440 nm. The optimization of the research was first established by varying the volume of sodium nitroprusside (0.5–7.0 ml), where we found maximum optical density at 5.5 ml; hence, it was coined as the optimized volume of reagent, as shown in Figure 3.

**Figure 4:**
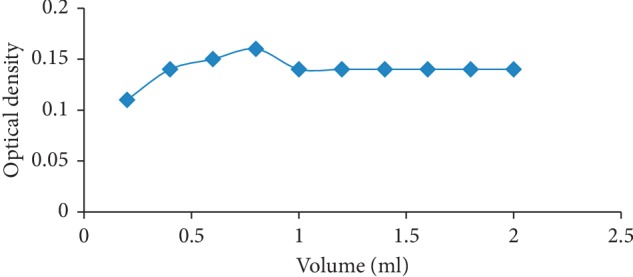
Volume optimization curve for 0.1% zinc chloride at 0.8 ml.

Similarly, the volume of 0.1% zinc chloride with maximum optical density was recorded, and the optimized volume was found to be 0.8 ml, as shown in Figure 4.

After optimizing sodium nitroprusside and zinc chloride separately, the optimized volumes of reagents are mixed with the drug to develop the color.

The optimum time for the completion of the reaction between raloxifene hydrochloride and sodium nitroprusside was 1 min, and the color was stable for 48 hours (the colored complex thus formed was stored up to 48 hours, and optical density of colored complex was measured), and it was quite stable with precise measurements. The concentration for measuring the colored complex was set at a higher level of the calibration curve, i.e., at 120 *μ*g·ml^−1^, to ensure the stability of the colored complex, as shown in Table 1.

### 3.1. Validation of Analytical Method for Raloxifene Hydrochloride

Validation of an analytical method is to establish that the performance characteristics of the developed method meet the requirements of the intended analytical application.

#### 3.1.1. Linearity

**Figure 5:**
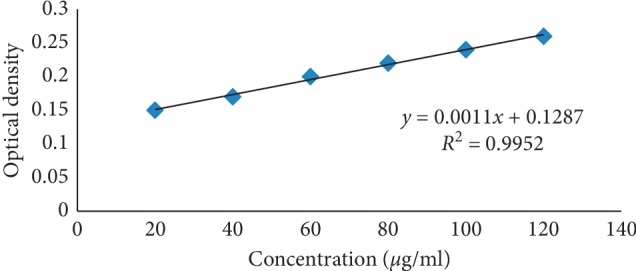
Calibration curve for raloxifene hydrochloride.

The colored complex of the drug with sodium nitroprusside and zinc chloride was analyzed using a colorimeter; the optical density of all aliquots (converted to colored complex) was taken to construct a calibration curve, as shown in Figure 5. The linearity was in compliance with the regression plot in the concentration range of 20–120 *μ*g/ml with a correlation coefficient (*R*^2^) of 0.995. Figure 5 shows the linear graph between concentrations of the drug complex and its optical density, as per the Beer–Lamberts law.

#### 3.1.2. Precision

The data for intraday and interday precision studies were obtained for three different concentrations 40, 80, and 120 *μ*g·ml^−1^ in linearity. The % RSD values for intraday and interday precision were less than 2, and the result is shown in Table 2.

#### 3.1.3. Accuracy

The accuracy of the method was evaluated in triplicate at three concentration levels, i.e., 80%, 100%, and 120%, of the target test concentration (50 *μ*g·ml^−1^ of raloxifene). The percentages of recoveries were calculated, and the results are presented in Table 3.

#### 3.1.4. Limits of Detection and Quantitation

The limit of detection (LOD) and limit of quantitation (LOQ) for the procedure were performed on samples containing very low concentrations of analytes under the ICH guidelines. By applying the mathematical formula, LOD was expressed by establishing the minimum level at which the analyte can be reliably detected. LOQ was considered as the lowest concentration of analytes in standards that can be reproducibly measured with acceptable precision. The LOD and LOQ values for raloxifene are shown in Table 4.

#### 3.1.5. Assay

The assay procedure was performed in triplicate, and the percentage of drugs found in the formulation, mean, and standard deviation in the formulation were calculated and are shown in Table 5.

## 4. Conclusion

The selected drug raloxifene was determined in the pure form by a colorimetric method, and this method was validated as per the ICH guidelines. The linearity range for raloxifene was 20–120 *μ*g/ml, with an *R*^2^ value of 0.995. The % RSD for intraday and interday precision was <2%. The method has been validated in an assay of the pure form. The developed method was compared with the already proposed methods, and we found it more reliable with all the parameters. While comparing with Annapurna et al.'s work where the color was developed with the aid of heating at 60° for 15 min, this method is devoid of heat and hence it is surely beneficial. Furthermore, Basavaiah et al. reported the accuracy in terms of recovery as 97%, whereas the proposed method leads in terms of accuracy. The accuracy of the method was validated by recovery studies and was found to be significant and under specification limits, with a % recovery of 100.5%. The assay result was found to be within the acceptable limits.

## Figures and Tables

**Table 1:** Stability of the colored complex of 120 *μ*g·ml^−1^ with respect to time (hours).

Time (hours)	Optical density
4	0.20
8	0.21
12	0.21
16	0.21
20	0.21
24	0.21
28	0.21
32	0.21
36	0.21
40	0.21
44	0.20
48	0.18

**Table 2:** Intraday and interday precision.

Concentration (*μ*g·ml^−1^)	Intraday (*n* = 3)		Interday (*n* = 3)	
	Amount of drug found (mean ± SD)	% RSD	Amount of drug found (mean ± SD)	% RSD
40	39.9 ± 0.115	0.288	39.16 ± 0.55	1.40
80	80.43 ± 0.763	0.948	78.16 ± 1.16	1.48
120	119.9 ± 0.173	0.144	119.3 ± 0.458	0.38

**Table 3:** Accuracy of raloxifene hydrochloride.

Name of the drug	Concentration of drug (*μ*g/ml)	Recovery level (%)	Amount of drug added (*μ*g/ml)	Amount found (*μ*g/ml) (mean ± SD)	% recovery (*n* = 3)
Raloxifene hydrochloride	50	80	40	40.2 ± 0.057	100.5
		100	50	49.8 ± 0.115	99.6
		120	60	60.2 ± 0.121	100.33

*n* = number of replicates; SD = standard deviation.

**Table 4:** LOD and LOQ value for raloxifene hydrochloride.

Parameter	Raloxifene
LOD (*μ*g/ml)	1.8074
LOQ (*μ*g/ml)	5.477

**Table 5:** Assay for raloxifene hydrochloride.

Formulation^a^	Labelled claim	Amount found (mean ± SD)	Assay (%)
Tablet 1	60 mg	59.85 ± 0.105	99.75
Tablet 2	60 mg	59.69 ± 0.194	99.48

^a^Tablet 1 and tablet 2 are two formulations from two different companies.

## Data Availability

The data used to support the findings of this study are included within the article.
